# Self-Control Modulates the Behavioral Response of Interpersonal Forgiveness

**DOI:** 10.3389/fpsyg.2020.00472

**Published:** 2020-03-27

**Authors:** Hui Liu, Haijiang Li

**Affiliations:** Department of Psychology, Shanghai Normal University, Shanghai, China

**Keywords:** self-control, interpersonal forgiveness, decision-making, cognitive process, prosocial behavior

## Abstract

Previous studies have shown that forgiveness is associated with the ability of self-control. However, whether self-control can modulate interpersonal forgiveness remains unclear. In the current study, we aimed to explore the relationship between self-control and the process of forgiveness using a behavioral measure of forgiveness during which participants distributed money between themselves and unknown others who had previously treated them fairly or unfairly in an adapted decision-making task. Seventy-two participants with low or high self-control were recruited based on their scores on the self-control scale (SCS). Results showed that participants exhibited increased anger and decreased happiness after experiencing unfair treatment. Participants with high self-control distributed more money to opponents who previously treated them unfairly compared with those with low self-control, whereas no such difference was observed to opponents who previously treated them fairly between the two groups. A significantly positive correlation was also found between the forgiveness rates and participants’ self-control scores. These findings suggest that self-control modulates interpersonal forgiveness responses. Individuals with high self-control expressed an increased prosocial response toward people who previously offended them, which is similar to the process of forgiveness.

## Introduction

Experiencing conflict, offense, or unfairness is inevitable in a social situation; forgiveness is regarded as a good approach to reduce these threats and increase harmony in society (Burnette et al., 2014). Psychological studies on forgiveness have been around for approximately 40 years; however, a consistent definition of forgiveness remains lacking (Berry et al., 2005; Worthington, 2007; Riek and Mania, 2012). Most researchers agree that interpersonal forgiveness is a transformation process of prosocial motivation (McCullough et al., 1997; McCullough, 2000), including the reduced motivation of retaliation and avoidance and increased benevolent motivation toward a transgressor (McCullough et al., 2000). In the current study, McCullough’s definition of forgiveness was used and considered as a changing process of prosocial motivation during which individuals choose a prosocial approach (e.g., forgiveness, mercy, and reconciliation) toward a perpetrator instead of retaliation or avoidance (McCullough et al., 1997; Worthington and Wade, 1999).

After being offended, the dominant response toward a perpetrator is anger, hostility, or revenge rather than forgiveness (Slotter et al., 2012; Civai, 2013; Gilam et al., 2019). When choosing to forgive, individuals have to overcome the influence of automated negative reactions (e.g., hostility and revenge), which requires the involvement of cognitive control or self-control (Pronk et al., 2010; Wilkowski et al., 2010; Maier et al., 2018). Self-control refers to one’s ability to consciously suppress impulsive, habitual, or automatic cognition, emotions, and reactions to achieve a specific goal (Baumeister et al., 2007; Muraven et al., 2007). Studies show that high self-control is associated with improved interpersonal relationships and suppression of their impulses and reactions (Baumeister and Heatherton, 1996; Vohs and Heatherton, 2000; Tangney et al., 2004; Cheung et al., 2014).

Previous studies have also explored the relationship between self-control and forgiveness. Research has indicated a positive correlation between self-control and forgiveness (Tangney et al., 2004; DeWall et al., 2010; Vohs et al., 2011; Pronk et al., 2019). Finkel and Campbell (2001) first explored the relationship between self-control and forgiveness in romantic relationships and found that self-control can predict individual differences of forgiveness. In a large behavioral survey of self-control, a moderately positive correlation between self-control and the tendency to forgive others was found (Tangney et al., 2004). DeWall et al. (2010) explored the relationship between self-control and forgiveness by combining the physiological indicators of self-control (i.e., the efficiency of the human body’s utilization of glucose). They observed that deficiency in glucose is related to a low tendency to forgive others and a low rate of cooperation. Research suggestes that the higher the self-control ability is, the better the relationship quality will be no matter whether it is between friends, lovers, or couples. Moreover, people with high self-control have a high tendency of forgiveness and relationship satisfaction, as well as the absence of conflict (Vohs et al., 2011). A recent meta-analysis study confirmed a small to moderate correlation between self-control and forgiveness, and a relationship was proven to became stronger when forgiveness was measured by low retaliatory motivation rather than high benevolent motivation (Burnette et al., 2014). Pronk et al. (2019) investigated the changes in self-control and forgiveness of newly married couples in the first 4 years of marriage. The results showed that the level of self-control and forgiveness gradually increased over time, and a positive concurrent correlation existed between them. Neuroimaging studies observed increased activation of the dorsolateral prefrontal cortex (DLPFC), which plays a vital role in cognitive control when overcoming unwanted negative emotional responses (Maier et al., 2018). Forgiving a transgressor is associated with elevated responses of DLPFC, which suggests that many cognitive control resources are needed to inhibit negative emotional responses toward a transgressor (Brüne et al., 2013).

In the present study, we aim to explore whether self-control can modulate interpersonal forgiveness using a behavioral measure of forgiveness. This study is the first to explore the relationship between self-control and interpersonal forgiveness using the behavioral measurement paradigm combined with adapted “ultimatum game” (UG) and “dictator game” (DG) to measure forgiveness. The behavioral measurement paradigm can better conceal the purpose of the experiment, obtain more real responses from participants, and avoid the limitation of self-report scales as a social expectation effect (Worthington et al., 2015; Li and Lu, 2018). In addition, the behavioral measure of forgiveness provides a new perspective to investigate the psychological process of forgiveness that questionnaires cannot achieve (McCullough et al., 2003; Dorn et al., 2014). In this behavioral task of forgiving response, two stages were included. The first stage was the adapted “UG” in which the participants would experience offense or unfair treatment and then observe the allocation proposal of participants made in the adapted “DG” as indexes of forgiveness or not. Previous studies have found that the behavioral measure paradigm of forgiveness can induce the same offensive feelings as in the real environment and has a significant positive correlation with individuals’ self-reported trait forgiveness (Sanfey et al., 2003; Harlé et al., 2010; Carlisle et al., 2012; Dorn et al., 2014; Gilam et al., 2019). On this basis, we made two hypotheses: (1) after completing the adapted UG, participants would express emotional fluctuation because of the unfair experience; and (2) participants with high self-control would give a more fair distribution to opponents who previously treated them unfairly than those with low self-control.

## Materials and Methods

### Participants

Two-hundred seventy participants were selected from a pool of undergraduate students at a university in China based on their scores on the 13-item brief self-control scale (SCS; Tangney et al., 2004; Tan and Guo, 2008; Unger et al., 2016). Scores for the SCS ranged from 21 to 61 (Cronbach’s α = 0.77). On the basis of a previous study (Tan and Guo, 2008), participants were selected for either the high self-control group (i.e., score on the SCS was in the highest 27%) or the low self-control group (i.e., score on the SCS was in the lowest 27%). The participants were randomly selected and voluntarily participated in the current study. According to the experimental design, the prior analysis shows that the sample size is 36 when the statistical power is 0.95 (Faul et al., 2007). In the current study, after removing eight participants who did not trust the cover story that they were playing with real opponents, the final high self-control group comprised 36 students (14 males, 22 females, average age = 20.50 years, *SD* = 1.03), whereas the low self-control group consisted of 36 students (14 males, 22 females, average age = 20.28 years, *SD* = 0.78). Chi-square tests of gender and self-control group showed that no significant interaction existed between the self-control group and gender (*x*^2^_(__1__)_ = 1.71, *p* = 0.19). In comparison with the low self-control group, the high self-control group reported significantly higher level of self-control (*t*_[__70__]_ = 14.54, *p* < 0.001, *d* = 3.47; low self-control group: *M* = 30.47, *SD* = 3.57; high self-control group: *M* = 45.08, *SD* = 4.86). This study was conducted in accordance with the recommendations of the SHNU Ethics Review Board. All subjects gave written informed consent in accordance with the Declaration of Helsinki. The protocol was approved by the SHNU Ethics Review Board.

### Behavioral Experiment

**FIGURE 1:**
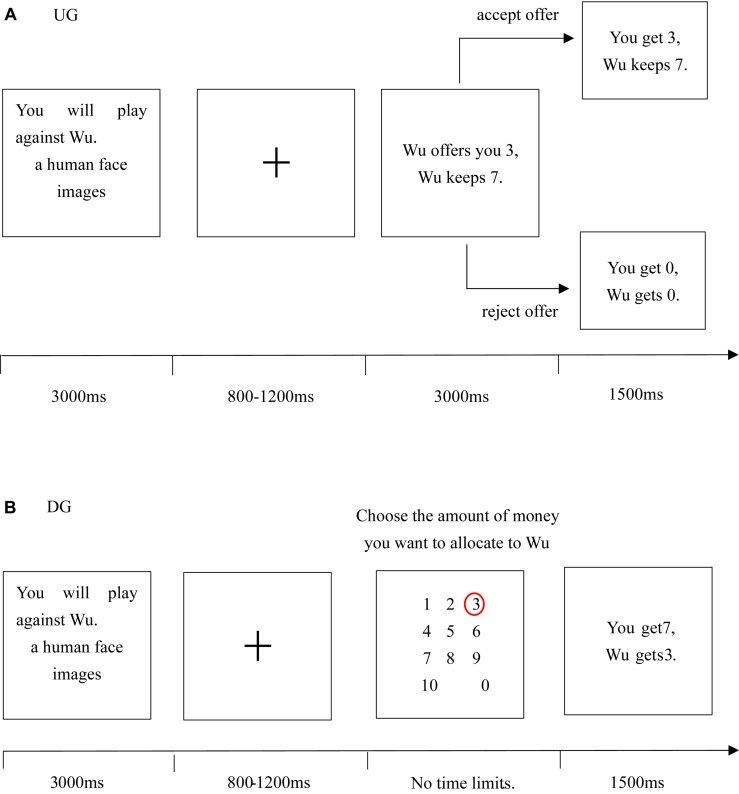
Schematic structure of behavioral experimental paradigm. The behavioral experimental paradigm consists of two stages: adapted UG **(A)** and DG **(B)**.

Before the experiment was conducted, the subjects were told that they would play a money allocation game online with other opponents, and their performance in the task would influence their final payment. The behavior experiment included two stages (Figure 1). In the first stage, the participants acted as the recipient against four other participants (i.e., two male and two female proposers) in an adapted UG. The proposer would allocate 10 RMB each trial and provide his distribution proposal, whereas the recipient (i.e., the subject) would choose to either accept or reject the proposer’s offer. If the offer was accepted, both sides would share the money according to the allocation proposal. Conversely, if the offer was rejected, both would not receive anything. In addition, two fair opponents of the four proposers (one male and one female) would consistently make relatively fair proposals (splits of 5:5; 4:6; and 3:7), whereas the other two unfair opponents (one male and one female) would consistently make relatively unfair proposals (splits of 2:8; 1:9; and 0:10). Hence, the implicit task in the UG was to identify whether the proposer was fair.

The specific experimental process of UG was as follows. First, participants saw the name and picture of their opponents (viewing period for 3000 ms), and this was followed by a jittered 800–1200 ms of anticipation period (random fixation “+” appears on the screen). Then, the opponent’s proposal was presented for 3000 ms in the decision-making period. During this period, the subjects had the option to accept or reject by pressing the “j” button for rejection, which would result in both sides receiving nothing, or the “f” button for acceptance, which would result in 10 yuan beeing split according to the offers. Finally, in the feedback phase for 1500 ms, the amount of money each side received appeared on the screen.

In the second stage, the subjects’ roles were reversed to be dictators (i.e., proposers), and participants were asked to distribute 10 RMB with four previous opponents (two fair opponents and two unfair opponents) in the DG each trial. The receiver had no right to refuse and could only passively accept the dictator’s proposal unlike in UG (Kahneman et al., 1986). If participants chose a fair distribution proposal (e.g., a split of 5:5) with their former unfair opponents, then they still acted kindly after being treated unfairly, which is similar to the performance of forgiving the former unfair opponents. Conversely, if participants chose an unfair distribution proposal (e.g., eight for themselves and two for opponents), then they were retaliating or punishing the former unfair opponents (Will et al., 2014, 2016). In addition, participants that chose to give a prosocial inequality allocation proposal (e.g., two for themselves and eight for opponents) to their opponent more than half of the sum (i.e., more than 5 RMB), which was regarded as a fair distribution, as suggested in a previous study (Maier et al., 2018).

The experimental process of DG is similar to UG. After the viewing period of 3000 ms and the anticipated period of jittered 800–1200 ms, the subjects were asked to decide how much money (0–10 RMB) to allocate to their previous fair or unfair opponents. Finally, the participants could see how much money they and their opponents had received in the feedback phase of 1500 ms. The experiment comprised 144 trials, 72 in UG, and 72 in DG. During the experiment, the interval between each trial was jittered 2000–3000 ms. All measures, conditions, and data exclusions were been reported. Details on each experimental condition can be found in the Supplementary Material.

### Experimental Procedure

First, 270 undergraduates were tested using the brief SCS. Subsequently, participants who met the requirements were invited to the laboratory for experiments according to their score (score in the highest 27% and the lowest 27%). Prior to the experiment, the subjects filled in the informed consent form and demographic information. Moreover, the subjects’ basic emotional states (e.g., anger, fear, happiness, and sadness) were measured using a five-point scale. Then, the subjects completed the adapted UG and re-evaluated their emotional states. Finally, participants completed the adapted DG as well as the measurement of the basic emotional states. Then, the subjects were asked how they felt about the experiment, some expressed doubts about the manipulation of the experiment and did not trust the cover story that they were playing with real opponents (eight subjects had been removed in the subsequent analyses). Thereafter, we explained the purpose and method of the experiment to the subjects, including the purpose of deceiving the real opponents of the subjects to obtain objective and real experimental results. Finally, the participants expressed their understanding and accepted payment of 25–30 yuan.

## Results

### Changes of Emotional States Before and After Each Stage

We conducted a repeated-measures ANOVA at three time points (i.e., baseline state, after UG, and after DG) for each emotional state to explore the changes of emotional states before and after each stage. The results showed that, after Phase 1, subjects felt more anger (*p* < 0.01, *d* = 0.49) and less happy (*p* < 0.05, *d* = 0.29) compared with the baseline state. In comparison with the baseline state, participants felt less fear (*p* < 0.05, *d* = 0.40) and less happy (*p* < 0.05, *d* = 0.32) after Phase 2. No other significant differences were found in other emotional states at different time points compared with other time points.

### Acceptance Rates of Unfair and Fair Offers in UG

**FIGURE 2:**
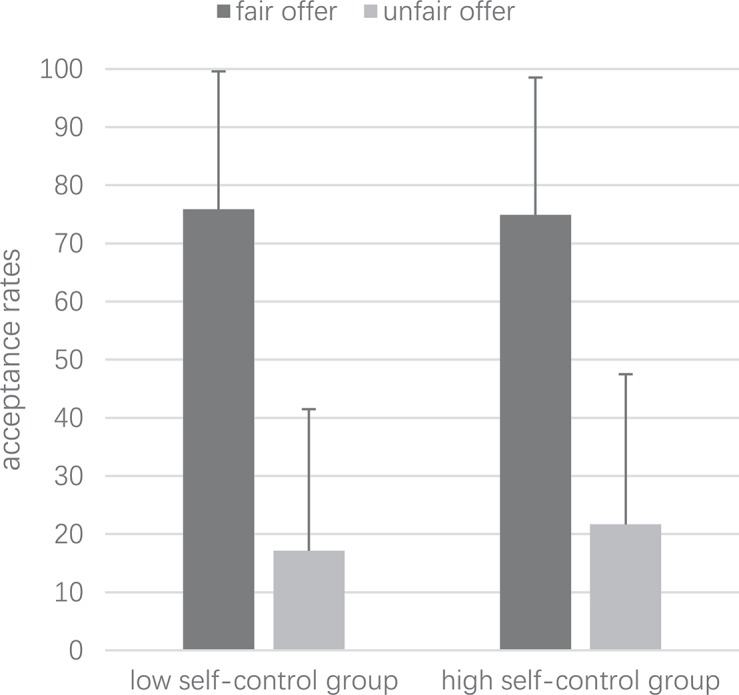
UG acceptance rates (%). The figure shows the acceptance rates of fair offer (dark area) and unfair offer (light area) for low self-control group (**left side of picture**) and high self-control group (**right side of picture**). Error bars represent positive standard errors.

Low self-control subjects accepted 75.84% fair proposals and 17.14% unfair proposals (Figure 2), whereas high self-control accepted 74.92% fair proposals and 21.68% unfair proposals (Figure 2). No significant difference was found between the high and low self-control groups in accepting fair proposals (*t*_[__70__]_ = 0.17, *p* > 0.05) and unfair proposals (*t*_[__70__]_ = −0.77, *p* > 0.05). However, participants in each group accepted more fair proposals than unfair proposals (low self-control: *t*_[__70__]_ = 10.36, *p* < 0.001, *d* = 2.44; high self-control: *t*_[__70__]_ = 9.13, *p* < 0.001, *d* = 2.15).

### Distribution Behavior in DG

**FIGURE 3:**
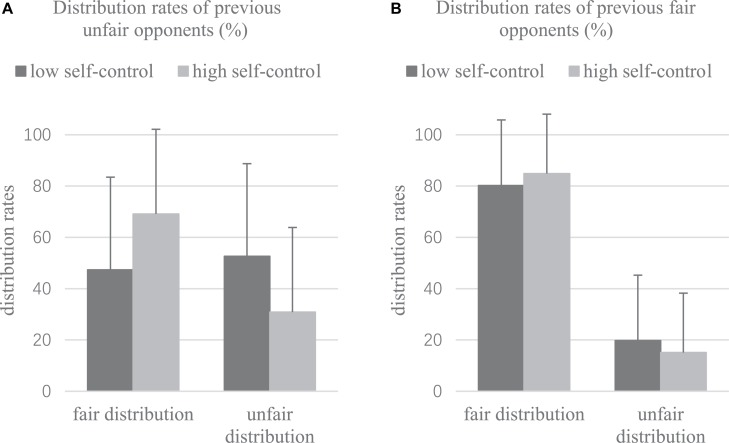
DG distribution rates (%). Two pictures show the allocation rates of subjects in the low self-control group (dark area) and the high self-control group (light area) to previous unfair opponents **(A)** and fair opponents **(B)**. The ratios between the number of trials representing fair distribution to previous fair opponents, unfair distribution to previous fair opponents, fair distribution to previous unfair opponents, and unfair distribution to previous unfair opponents as well as the total number of trials presented in DG are calculated. Error bars indicate positive standard errors.

The results showed that for the former unfair opponents, the rate of fair distribution was 47.38% in the low self-control group and 52.62% in the unfair distribution, whereas the rate of fair and unfair distribution in the high self-control group was 69.14% and 30.86%, respectively (Figure 3A). However, for the former fair opponents, the low self-control group had 80.25% fair distribution and 19.75% unfair distribution, whereas the high self-control group had 84.88% fairness and 15.12% unfairness (Figure 3B). We also conducted a 2 × 2 × 2 repeated-measures ANOVA with opponents (classified according to the offer in UG as formerly fair vs. formerly unfair) and allocation proposal (fair vs. unfair) as within-subject factors, self-control group (low self-control vs. high self-control) as a between-subject factor, and distribution rates selected in DG as a dependent variable. The results showed a significant main effect of allocation proposals (*F*_[__1_,_70__]_ = 49.484, *p* < 0.001, η*^2^* = 0.414) and a significant interaction effect of allocation proposals × self-control groups (*F*_[__1_,_70__]_ = 5.171, *p* < 0.05, η*^2^* = 0.069) and opponents × allocation proposals (*F*_[__1_,_70__]_ = 36.839, *p* < 0.001, η*^2^* = 0.345). A simple effect analysis showed that subjects in the high self-control group made more fair distributions (*t*_[__70__]_ = −2.28, *p* < 0.05, *d* = 0.54) and fewer unfair distributions (*t*_[__70__]_ = 2.28, *p* < 0.05, *d* = 0.54) than subjects of the low self-control group. Moreover, a significant three-factor interaction of opponents × allocation proposals × self-control groups (*F*_[__1_,_70__]_ = 4.574, *p* < 0.05, η*^2^* = 0.061) was observed. Individuals with high self-control carried out more fair distributions (*p* < 0.01) and fewer unfair distributions (*p* < 0.01) to the previous unfair opponents compared with those with low self-control. However, no significant difference was found between the allocation proposals of the high and low self-control groups to the previous fair opponents (both *ps* > 0.05).

### Correlation Between Forgiveness Rate and Self-Control

To further explore the relationship between self-control and interpersonal forgiveness, we examined the correlation between self-control scores and forgiveness rates (fair distribution proposals to former unfair opponents) and retaliation rates (unfair distribution proposals to former unfair opponents). The results showed a significant positive correlation between self-control and forgiveness rate (*r*_(__72__)_ = 0.26, *p* < 0.05) and a significant negative correlation with retaliation rate (*r*_(__72__)_ = −0.26, *p* < 0.05).

## Discussion

In this study, we aimed to explore the relationship between self-control and interpersonal forgiveness. An adapted economic decision-making task was used to simulate the process of interpersonal forgiveness, and the self-reported SCS was used to measure individuals’ ability of self-control. The results are consistent with our hypotheses that individuals with high self-control make more fair distribution toward opponents who previously treated them unfairly than those with low self-control. This result suggests that individuals with high self-control give a more forgiving response toward opponents who previously offended them than those with low self-control. We also found that the subjects felt more anger and less happiness after experiencing unfair treatment during the adapted UG. These findings suggest that self-control modulates interpersonal forgiveness.

The results show that after the participants completed the adapted UG, they felt more anger and less happy than the emotional states at the baseline. This result is consistent with previous research using the UG, which has shown that UG can induce the negative feelings of the subjects and let the subjects experience similar feelings after being offended in an actual situation (Sanfey et al., 2003; Harlé et al., 2010; Gilam et al., 2019). In UG, when subjects encountered an unfair opponent’s proposal, they feel that they were offended or unfairly treated and that their self-interest was damaged, which caused negative emotions (Sanfey et al., 2003). Similarly, Carlisle et al. (2012) found that subjects in offense conditions felt less positive and more negative emotions after experiencing the unequal distribution of raffle tickets in Round 1 compared with the subjects in the no offense condition, which was consistent with the feelings experienced by participants during UG. In addition, the subjects’ happiness decreased after DG compared with the emotional states at baseline. This result may be due to the offended experience of the UG process and the difficulty in returning to the baseline level of happiness.

In UG, the subjects in high and low self-control groups accepted more fair distributions compared with unfair distributions. Thus, the subjects could realize the two situations of fair and unfair distributions in UG, and they tended to pursue fairness and equity (Trivers, 1971; Fehr and Fischbacher, 2004); thus, fair distributions were more accepted. This finding can be confirmed by previous studies that found similar results (Sanfey et al., 2003; Brüne et al., 2013; Maier et al., 2018). Moreover, a small number of unfair distributions were accepted, that is, most of the unfair distributions were rejected, which may be due to individuals’ pursuit of fairness, even if it damaged their own interests.

The relationship between self-control and interpersonal forgiveness could be observed from the participants’ allocation proposals toward opponents who previously treated them unfairly in the DG. In line with our hypothesis, the participants with high self-control carried out a more fair distribution to opponents who treated them unfairly than those with low self-control. This result suggests that participants with high self-control tended to give a forgiving response to previously unfair opponents more than those with low self-control; however, no differences were observed between the two groups in terms of previous fair opponents. Moreover, a positive correlation was observed between self-control scores and forgiveness rates. These findings were guaranteed by previous studies that found that self-control could predict interpersonal forgiveness (Tangney et al., 2004; Vohs et al., 2011; Burnette et al., 2014; Pronk et al., 2019). Self-control is positively related to interpersonal forgiveness (Finkel and Campbell, 2001; Bal Balliet et al., 2011), and the correlation between self-control and forgiveness is strong when forgiveness is measured with low retaliation rather than high benevolence (Burnette et al., 2014). A recent longitudinal study on self-control and forgiveness in marriage found a positive correlation between self-control and forgiveness, and, over time, married individuals have been proven to have more self-control and become more forgiving (Pronk et al., 2019). In addition, individuals with high self-control are more willing to forgive offenders (Tangney et al., 2004; Vohs et al., 2011). When individuals are offended, the first reaction is destructive and the emotions are negative (e.g., anger and retaliation) to safeguard their own interests (Righetti et al., 2013). Self-control is regarded as an important ability that enables individuals to shift from caring for their own interests to caring for more values and considerations (i.e., pro-relationship behavior) (Finkel and Campbell, 2001). Increased self-control makes individuals have greater potential to suppress the destructive impulse after deep consideration and show a constructive behavior, such as forgiving offenders (Finkel and Campbell, 2001; Hofmann et al., 2009; Pronk and Righetti, 2015). Moreover, self-control can help individuals suppress the impulse to retaliate by reducing the rumination of offensive events (Pronk et al., 2010). A neuroimaging study also found that granting forgiveness toward previously unfair opponents elicits increased activation of the DLPFC, a brain region mainly responsible for cognitive control (Brüne et al., 2013; Maier et al., 2018). These findings suggest that when individuals are offended or treated unfairly, high self-control may help them suppress prevailing negative emotions caused by unfair treatment and promote prosocial behavior, such as forgiveness.

This study has some limitations. One is that some subjects questioned the authenticity of economic decision-making tasks, which may affect the experimental results. Thus, these subjects were excluded from the subsequent analyses. Moreover, this study repeated previous research findings and found that self-control could modulate interpersonal forgiveness. Nevertheless, different behavioral measurement paradigms for measuring forgiveness involve different forgiveness-related psychological processes, and future research requires the use of other behavioral measurement paradigms to verify the relationship between self-control and interpersonal forgiveness. Third, participants were not required to report their intention when giving fair distribution toward a prior unfair opponent, which makes the forgiving conclusion negotiable. However, we found no significant difference in anger levels after DG between the high and low self-control groups (*t*_[__70__]_ = 1.727, *p* > 0.05). The measurement and analysis of anger can reveal whether the subjects’ behaviors are due to forgiveness or forbearance. Additionally, the finding helps us ensure that participants forgive their previous unfair opponents to some extent. Future research needs to collect self-report data when using the behavioral paradigm of forgiveness. Finally, our study was a cross-sectional one, and we did not manipulate self-control; thus, the causal relationship between self-control and interpersonal forgiveness could not be determined. Future studies may use longitudinal research or manipulate self-control to examine the causal relationship between self-control and interpersonal forgiveness.

In sum, the current study extends previous findings concerning self-control and interpersonal forgiveness and provides a different perspective of behavioral measure to further explore the relationship between self-control and interpersonal forgiveness. This study has been the first to explore the relationship between self-control and interpersonal forgiveness using the behavioral measurement paradigm combined with adapted UG and DG. Our findings suggest that, when confronted with interpersonal conflicts, individuals with high self-control ability could suppress the negative emotions generated by their instincts and show increased prosocial behaviors consistent with their long-term goals toward transgressor, such as forgiveness.

## Data Availability Statement

The datasets generated for this study are available on request to the corresponding author.

## Ethics Statement

The studies involving human participants were reviewed and approved by the Shanghai Normal University Ethics Review Board. The patients/participants provided their written informed consent to participate in this study.

## Author Contributions

HJL and HL conceived and designed the experiments. HL collected the data and performed the statistical analysis. HJL and HL contributed to the writing of manuscript.

## Conflict of Interest

The authors declare that the research was conducted in the absence of any commercial or financial relationships that could be construed as a potential conflict of interest. The handling Editor is currently editing co-organizing a Research Topic with one of the author, HJL and confirms the absence of any other collaboration.
