# Supplementary material for: Self-Control Modulates the Behavioral Response of Interpersonal Forgiveness
Source: Front Psychol. 2020 Mar 27;11:472. doi: 10.3389/fpsyg.2020.00472 (PMC7118213; doi:10.3389/fpsyg.2020.00472)
Supplement: Supplementary file 1 [file Table_1.DOCX]

|  | weight | opponents’ name | opponents’ gender | fair or unfair | opponents’ advice |
| --- | --- | --- | --- | --- | --- |
| 1 | 6 | Li | male | fair | Li offers you 5, Li keeps 5. |
| 2 | 6 | Li | male | fair | Li offers you 4, Li keeps 6. |
| 3 | 6 | Li | male | fair | Li offers you 3, Li keeps 7. |
| 4 | 6 | Zhao | female | fair | Zhao offers you 5, Zhao keeps 5. |
| 5 | 6 | Zhao | female | fair | Zhao offers you 4, Zhao keeps 6. |
| 6 | 6 | Zhao | female | fair | Zhao offers you 3, Zhao keeps 7. |
| 7 | 6 | Wu | male | unfair | Wu offers you 2, Wu keeps 8. |
| 8 | 6 | Wu | male | unfair | Wu offers you 1, Wu keeps 9. |
| 9 | 6 | Wu | male | unfair | Wu offers you 0, Wu keeps 10. |
| 10 | 6 | Han | female | unfair | Han offers you 2, Han keeps 8. |
| 11 | 6 | Han | female | unfair | Han offers you 1, Han keeps 9. |
| 12 | 6 | Han | female | unfair | Han offers you 0, Han keeps 10. |

the advices from each of the fair and unfair opponents

Note. 72 opponents’ proposals were presented in random order during the experiment.
